# Inoculum composition determines microbial community and function in an anaerobic sequential batch reactor

**DOI:** 10.1371/journal.pone.0171369

**Published:** 2017-02-14

**Authors:** Allison R. Perrotta, Rajkumari Kumaraswamy, Juan R. Bastidas-Oyanedel, Eric J. Alm, Jorge Rodríguez

**Affiliations:** 1 Department of Civil and Environmental Engineering, Massachusetts Institute of Technology (MIT), Cambridge, MA, United States of America; 2 Department of Chemical and Environmental Engineering (CEE), Masdar Institute of Science and Technology, Abu Dhabi, UAE; 3 Department of Biological Engineering, MIT, Cambridge, MA, United States of America; 4 Broad Institute of MIT, Cambridge, MA, United States of America; Colorado State University, UNITED STATES

## Abstract

The sustainable recovery of resources from wastewater streams can provide many social and environmental benefits. A common strategy to recover valuable resources from wastewater is to harness the products of fermentation by complex microbial communities. In these fermentation bioreactors high microbial community diversity within the inoculum source is commonly assumed as sufficient for the selection of a functional microbial community. However, variability of the product profile obtained from these bioreactors is a persistent challenge in this field. In an attempt to address this variability, the impact of inoculum on the microbial community structure and function within the bioreactor was evaluated using controlled laboratory experiments. In the course of this work, sequential batch reactors were inoculated with three complex microbial inocula and the chemical and microbial compositions were monitored by HPLC and 16S rRNA amplicon analysis, respectively. Microbial community dynamics and chemical profiles were found to be distinct to initial inoculate and highly reproducible. Additionally we found that the generation of a complex volatile fatty acid profile was not specific to the diversity of the initial microbial inoculum. Our results suggest that the composition of the original inoculum predictably contributes to bioreactor community structure and function.

## Introduction

Research in wastewater treatment, traditionally focused on pollutant removal, has expanded to include sustainable recovery of resources such as biofuels and organic acids from waste streams. Valuable products such as methane, hydrogen, solvents and bio-plastics can be generated from wastewater streams by microbial fermentation [1–6]. However, the large-scale deployment of bioreactors for resource recovery purposes is limited due to persistent variability of the chemical profiles produced [7]. Most research focusing on product variability from microbial fermentations focuses on how abiotic operational conditions of bioreactors affect production and stability. In parallel, many environmental microbiologists focus on the role of community assembly in defining complex microbial community structure and function [7–13]. Yet these two complementary fields show minimal overlap in the literature. There are other examples in the literature in which the role that the initial microbial inoculum plays in bioreactor production has been investigated. But, these works have focused on other product profiles, specifically methane production, and have infrequent sampling of the microbial community [13, 14].

Previous studies have identified a correlation between high diversity and functional redundancy within complex microbial communities. This work has lead to an emphasis in inoculum diversity over composition [15–17]. However, many of the studies demonstrating this correlation focused upon broad ecosystem functions such as respiration and biomass and not upon the production of a desired chemical profile [15–17]. Additionally, microbial successional dynamics have been shown to play a large role in initiating bioreactors and can greatly affect community predictability, diversity, and the complexity of the product profile [9,10,13,17].

We sought to test how initial microbial inocula derived from different sources affect microbial community structure and ecosystem function with a focus on producing a complex and even profile of volatile fatty acids (VFAs). Identical sequential batch reactors (SBRs) were inoculated with three naturally occurring sources for microbial inocula: camel manure (Camel), mangrove sediment (Mangrove), and wastewater treatment sludge (Sludge). Experiments were conducted in triplicate and abiotic operational conditions favored the fermentative production of VFAs; specifically, dark anaerobic fermentation where methanogenesis is inhibited via low pH [6]. Glucose was the sole carbon source provided throughout the experiment. Reactors underwent sequential batch cycles every 48 hours to ensure a steady supply of glucose as well as limit product inhibition and contamination [18]. Chemical production and microbial composition of each reactor were tracked during a 14-day fermentation period.

## Materials and methods

### Inocula and fermentation

Camel manure from Al Ain camel market, mangrove intertidal sediment (less than 30 cm in depth) from Abu Dhabi coast (24° 31’45.5” N 54° 33’21”E), and anaerobic sludge from Al Mafraq wastewater treatment plant (Abu Dhabi Sewerage Services Company, Al Dhafrah, Abu Dhabi, UAE) served as starting inocula. These three sources were chosen as they represent three distinct environments and thus would provide very distinct yet highly diverse microbial communities. All sample collections were obtained from locations that are open to the public and did not involve any endangered or protected species.

Inocula were stored at 4°C for less than 5 days prior to the start of experiments (S1 Text). Fermentations were carried out at 37°C in 150 mL serum bottles with a working volume of 60 mL. Anaerobic serum bottles were loaded with media (see below for composition) and respective inoculum. The initial biomass concentration for all the three inocula was 10 g/l, measured as dry weight matter.

Sterile media consisted of the following components: glucose 5 g/L, phosphate buffer Na_2_HPO_4_·2H_2_O 0.2 g/L and KH_2_PO_4_ 2.5 g/L diluted in basal anaerobic media [19], NH_4_Cl 1 g/L, NaCl 0.1 g/L, MgCl_2_·6H_2_O 0.1 mg/L: CaCl_2_·2H_2_O 1.2 g/L, (NH_4_)_6_Mo_7_O_24_·4H_2_O 0.05 g/L, CoCl_2_·6H_2_O 0.05 g/L, FeCl_2_·4H_2_O 2 g/L, MnCl_2_·4H_2_O 0.05 g/L, NiCl_2_·6H_2_O 0.1 g/L, Na_2_SeO_3_·5H_2_O 0.1 g/L, H_3_BO_3_ 0.05 g/L, CuCl_2_·H_2_O 0.04 g/L, ZnCl_2_ 0.05 g/L, AlCl_3_ 0.05 g/L, EDTA 0.5 g/L. Media pH was adjusted to 5.5 with HCl 1 M to suppress methanogenic activity. The glucose solution was autoclaved separately from the mineral media.

Reactors were inoculated by mixing inocula (Camel, Mangrove, or Sludge), mineral media and glucose solution. After the initial inoculation step, the glucose present in the media was the only carbon source provided. Following inoculation, each bottle was crimped using sterile rubber stoppers, and flushed with pure N_2_ for 2 minutes, in order to achieve anaerobic conditions. Flushing was performed using sterile filters, 0.45 μm pore size (Corning^®^, Corning, NY, USA), and syringes (BD^®^,San Jose, CA, USA).

**Fig 1:**
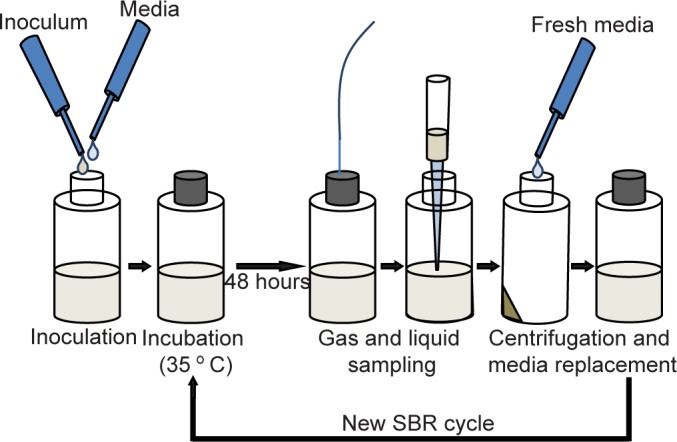
Sequential Batch Reactor (SBR) cycling was performed. SBR cycles involved centrifuging microcosms, for 20 minutes at 4,000 g, removal of the supernatant, and addition of fresh media. A total of 7 SBR cycles were performed per reactor.

All reactors were incubated without agitation in a temperature-controlled incubator, at 35°C for a total of 14 days. Three replicate reactors were utilized per inoculum source, which amounted to nine reactors being simultaneously incubated. Gas and liquid sampling, followed by SBR cycling (Fig 1), were performed every 48 hours. A total of 72 samples were collected consisting of baseline (cycle 0) and seven subsequent 48-hour time points (cycles 1–7). Each SBR cycle involved centrifuging the microcosms for 20 minutes at 4,000 g, removal of supernatant and addition of fresh media (Fig 1). After SBR cycling, the bottles were again crimped and flushed with N_2_. All inoculation, liquid sampling, and media addition/removal steps were performed in a UV sterilized laminar flow chamber. Liquid sampling was performed using sterile needles and consisted of collecting 3 ml from the broth for DNA extraction and liquid chemical analysis. Rubber stoppers were cleaned with 70% ethanol prior to liquid and gas sampling.

### Chemical analysis

Gas production was measured by liquid volume displacement. The liquid consisted of a saturated solution of NaCl (3 M) at pH 2 [20]. Chemical production of VFAs was detected by UV (210 nm) and quantified using an Agilent 1260 Infinity HPLC, Hi-Plex H 300 x 7.7 mm column. The column temperature was 65°C, with an eluent flow rate of 0.6 mL/min, the eluent consisted of 5 mM H_2_SO_4_. Glucose and ethanol were detected by refractive index detection at an optical unit temperature of 35°C.

### DNA extraction and amplicon based Illumina sequencing of 16S rRNA genes

DNA was extracted from the initial inocula and the bioreactor microbial communities using the Ultra Clean Soil DNA isolation kit (MOBIO Laboratories, Carlsbad, CA, USA) according to the manufacturer’s procedure. Paired-end Illumina sequencing libraries were constructed using a two-step PCR approach targeting 16S rRNA genes previously described by Preheim *et al*. [21].

All paired-end libraries were multiplexed into one lane and sequenced with paired end 150 bases on each end on the Illumina MiSeq platform at the Biomicro Center (MIT, Cambridge, MA).

### Sequencing data processing

Raw data were quality filtered using QIIME (version 1.3.0) [22]. Fastq files from the forward and reverse reads were processed using the split_library_fastq.py program of QIIME. After quality filtering, a total of 4,825,857 sequence reads were generated with an average of 68,930 per sample (±61 056, std. dev., n = 72).

Sequences were truncated when more than one base in a row dropped below a Phred quality score of 14, corresponding to a probability of error around 3.98%. Only sequences at least 99 bps long after quality filtering was retained. After quality-filtering, 16S rRNA universal primer sequences were removed from all reads, Operational Taxonomic Units (OTUs) were assigned using distribution-based clustering with default parameters, and singletons were removed using methods described in Preheim, *et al*. [21] (S2 Text).

### OTU analysis

After initial processing for quality, a total of 2,736 OTUs were identified and their relative abundances were assessed. 83 OTUs were identified as having a relative abundance of at least 1% across all samples; the majority of these being present only in the original inocula (cycle zero). 11 OTUs were identified with a significant abundance, greater than or equal to 1%, in samples collected during cycles 1 to 7. The taxonomic classification of all OTUs was determined using the Ribosomal Database Project (RDP) with a support cut-off of 0.5 [23] and were confirmed using BLAST (see above).

The analysis package QIIME was used to filter the OTU table by input inoculate and remove samples with less than a thousand read counts from further processing [24] (S3 Text). Two samples, M2.1 (Mangrove, second replicate first cycle) S1.6 (Sludge, first replicate sixth cycle), were removed due to low read count. As a result, a total of 70 samples were considered for downstream analyses.

Alpha (Shannon diversity) and beta (Jensen-Shannon distance) diversities [25] were calculated using pysurvey (S4 Text). The effective number of species, of order 1, represented in each sample was calculated by taking the inverse of the natural log of the calculated Shannon entropy.

Phylogenetic trees were produced for organization and visualization purposes (Fig 1) using unique OTU representative sequences. Prior to building a phylogenetic tree, QIIME was used to align OTU unique sequences to full-length 16S reference sequences in the greengenes database (version gg_13_5) [26]. The aligned sequences were trimmed of gaps using trimAL (version 1.2) [27] and a constraint file created from the RDP taxonomic classifications was generated using a custom Python script (version 2.7.10). FastTree [28] was then used to build the phylogenetic tree (S5 Text). Abundance heat maps associated with the phylogenetic trees were generated using Interactive Tree of Life [29].

### Statistical analysis

To assess the significance of differences in chemistry and microbial composition as well as that of replicate variance between the inoculum sources, multivariate analyses were performed using the vegan package in R (S6 Text). Distance matrices for these analyses were calculated using Euclidean and Jensen-Shannon distance [25] for chemistry and microbial data, respectively. Due to the lack of independence in measurements across cycles, these analyses were performed within and not across each cycle. Significance was defined as a p-value less than or equal to 0.05 for both forms of analysis.

## Results and discussion

### Environmental selection contributes to community structure

In the course of this work we sought to compare the impact of initial inoculum upon microbial community structure and ecosystem function within a bioreactor. Our ecosystem function of interest focused on producing a predictable and stable profile of VFAs. It was important to assess if there was selection acting upon the microbial communities due to the abiotic operational conditions of the bioreactors over the course of the experiment. We hypothesized that the shared and consistent abiotic conditions of the bioreactors, for example pH and retention time, should serve as shared selective pressures and lead to similarities across all communities. Thus we expected the common abiotic pressures in our system to result in selection of similar OTUs, decreased divergence over time, and reproducibility across replicates.

**Fig 2:**
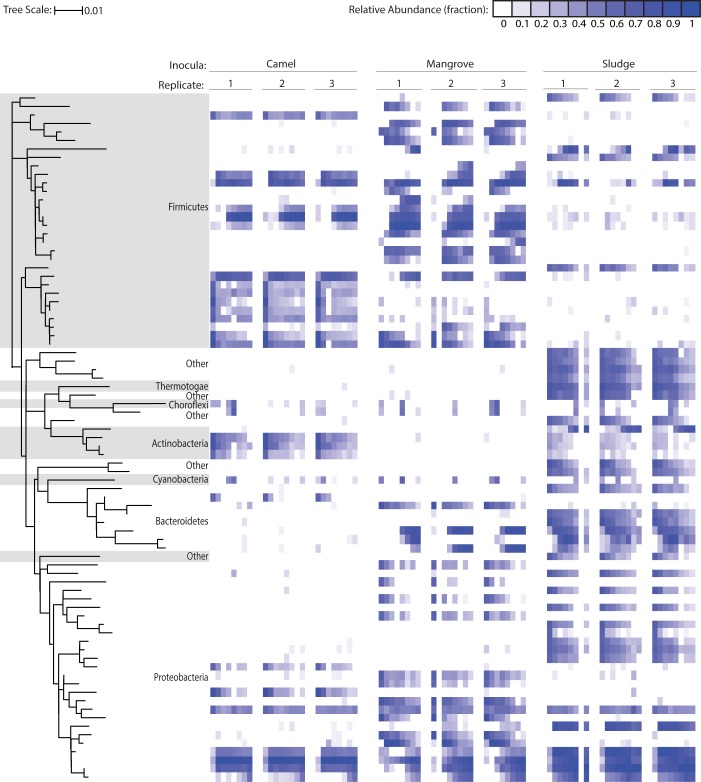
Microbial communities are reproducible and distinct to the initial microbial inoculum. **Relative** abundances of OTUs are represented as log relative abundances. Only OTUs that were present at equal or greater than 1% across all time points have been included. OTUs are organized vertically by phylogenetic relationship and horizontally by time point within a replicate, cycle 0 to cycle 7.

**Fig 3:**
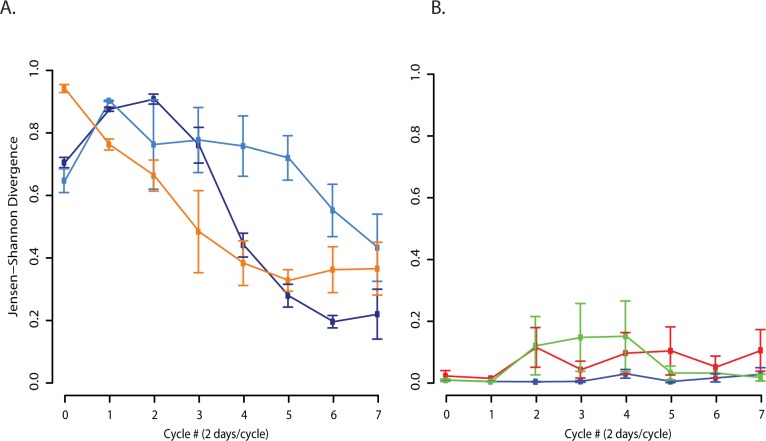
Divergence between reactors decreases overtime but remains low between replicates of the same inoculum. **A)** Average pairwise Jensen-Shannon divergence (family level) between Mangrove and Camel (dark blue), Mangrove and Sludge (light blue), and Sludge and Camel (orange) bioreactors. **B)** Average pairwise Jensen-Shannon divergence (family level) between Camel (blue), Mangrove (red), and Sludge (green) replicate bioreactors.

Abiotic selection pressures had consistent effects upon the microbial communities and confirm the reproducibility of our experiments. An enrichment of common OTUs was observed in all bioreactors (Fig 2 and S1 Fig). An overall decrease in pairwise divergence between communities was also observed. As seen in Fig 3A, pairwise comparisons of all microcosms consistently decreased in divergence over time, as measured by Jensen-Shannon distance [25]. The phylogenetic structure of replicates was also reproducible as evidenced by low divergence between replicates over time (Fig 3B). These conclusions are supported by recent work by Vanwonterghem *et al*. [13] in which microbial community structure within a methanogenic anaerobic digester was shown to be predictable and reproducible over an extended period of time.

**Fig 4:**
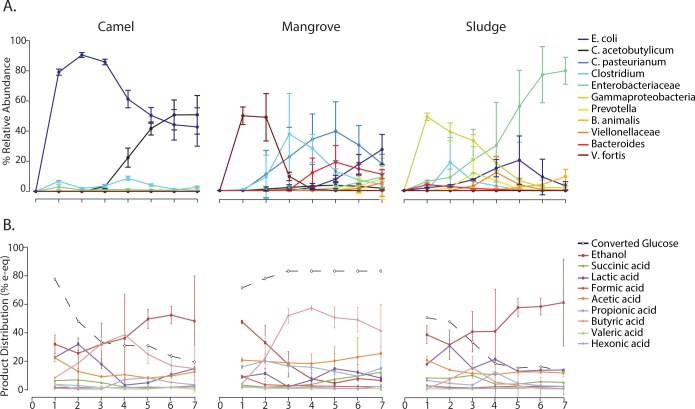
Dynamics of enriched OTUs and reactor chemical profiles are distinct to initial microbial inoculum. We illustrate changes in average relative abundances of enriched OTUs over time **(A)** and the changes in average product yields in terms of electron equivalents over glucose consumed from provided feed **(B)** for the three inocula sources Camel, Mangrove, and Sludge reactors, respectively.

Unsurprisingly, the observed enrichment represented by the most abundant OTUs, consisted of species known to produce hydrogen and VFA products in both mono- and mixed-culture bioreactors. More specifically, all bioreactors were enriched for *Clostridium pasteurianum*, *Clostridium acetobutyricum*, *Escherichia coli*, as well as other OTUs classified within the *Clostridium* and *Enterobacter* genera (Fig 4A) [1,18,30–32]. All enriched community members, with the exception of the *C*. *pasteurianum*, were present at very low abundances in at least two out of three replicates at cycle zero. *C*. *pasteurianum* was only detectible in one Camel replicate at time point zero. The abundance of these OTUs at cycle zero indicates that these community members were present at low relative abundances in the inoculum and were enriched for in our system.

These results suggest that the total diversity available for selection is extremely large, and underestimated even by deep 16S rRNA gene amplicon sequencing (S1 Fig). However, even in the light of the similar microbial enrichment observed in all bioreactors there were differences in the evenness and diversity of the microbial communities that were distinct to the microbial inoculum (Fig 4). This implies that even if diversity is large and shaped by abiotic selection, it may still fall short of the Bass-Becking “everything is everywhere” concept [33,34] in which the effects of initial inoculum on final community structure is negligible. Thus we tested the relative importance of inoculum on community structure.

### Microbial inoculum governs community structure and dynamics

While broad commonalities between bioreactor communities were observed they were overwhelmed by the effect of inoculum. At the community level, each inoculum generated a phylogenetic structure that was strikingly distinct and highly reproducible (Fig 2 and S2 Fig). The dynamics of individual abundant OTUs over time were highly dependent on the starting inoculum supplied (Fig 4A) and highly reproducible across replicates with in each of the inoculum sources (S3 Fig). Within each cycle, the microbial community in the bioreactors was significantly different between each inoculum group and the variance between replicates was insignificantly different, with the exception of cycle 6, as determined by multivariate analysis (S1 Table).

In Mangrove bioreactors, successional dynamics were observed and the final community contained OTUs belonging to *E*. *coli*, *C*. *pasteurianum*, *Enterobacteriaceae*, *Clostridium*, *Bacteroides*, and *Viellonellaceae*. Within this final community no single OTU made up more than 27% average relative abundance. Camel bioreactors underwent very little community succession, and the final community was dominated by two OTUs, *E*. *coli* and *C*. *acetobutylicum*, that were present at 47.2% and 50.8% average relative abundance respectively. In the Sludge bioreactors microbial succession was observed, but a single OTU belonging to the *Enterobacteriaceae* genus dominated the final cycles at 79.5% average relative abundance.

Many large environmental surveys have demonstrated that both biotic and abiotic processes shape community assembly in deterministic and stochastic ways and that their relative importance can vary greatly based on environmental pressures [35–37]. Our results indicate that inoculum source deterministically contributes to shaping community structure within an anaerobic SBR system.

### Microbial inoculum governs chemical composition

In addition to having community profiles that are reproducibly distinct, each inoculum was reproducibly predictive of bioreactor production over the two-week period of the experiment. Much like the observed community dynamics, chemical profiles were distinct to starting inoculum (Fig 4B). Within each cycle, the chemical profile of the bioreactors was significantly different between each inoculum group and the variance between replicates was insignificantly different, as determined by multivariate analysis (S2 Table).

The results of these experiments suggest that the functional capabilities of these communities arise in a deterministic fashion that is dependent upon starting inoculum.

Mangrove reactors maintained a high level of glucose conversion and a complex product profile throughout the experiment. These reactors exhibited a short adaptation period, evident due to a high production of ethanol (35% of all the products synthesized) during the initial cycles, followed by production of the desired products acetic, butyric, and propionic acids. The chemical profile of Mangrove reactors shifted to butyric acid production, up to 50%, in later cycles. Acetic and propionic acid as well as minor lactic acid production are observed throughout all cycles (Fig 4B).

In contrast to the Mangrove reactors, Camel and Sludge reactors demonstrated low glucose conversion over the course of the experiment. Glucose conversion in Camel microcosms dropped by almost half after the first cycle. By the final time point, ethanol made up more than 40% of all chemical products, and up to 79% of the glucose remained unconverted. This chemical profile indicates a loss of the desired function (Fig 4B). Sludge microcosms also failed to produce the desired fermentation profile in this experiment. In Sludge reactors, 30% of the provided glucose remained unconverted in the first cycle and up to 80% remained unconverted in later stages. The main products synthesized were ethanol and acetic acid (Fig 4B).

**Fig 5:**
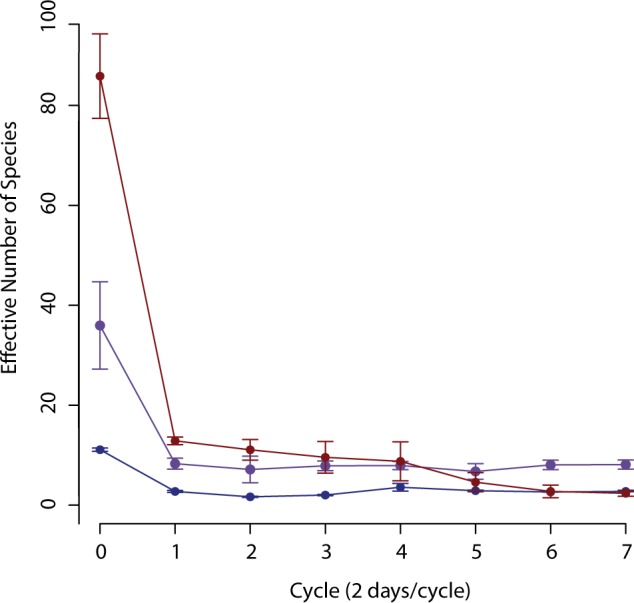
Initial inoculum alpha diversity is not correlated with reactor diversity. The microbial inocula used vary in diversity. The diversity of the microbial communities present in the reactors decreases over the course of the experiment. Diversity is presented as the effective number of species over time for Camel (dark blue), Mangrove (purple), and Sludge (red) bioreactors.

While the reasons for the difference in production between inoculum remain unclear, differences in the production potential of the community that are distinct from initial inoculum diversity could play a role. The initial Mangrove inoculum did not have the highest diversity of the three inoculum used (Fig 5). However, the community selected for in Mangrove reactors maintained a higher diversity and evenness throughout the experiment (Fig 5) and produced a complex chemical profile (Fig 4B). Sludge and Camel, demonstrating the highest and lowest initial diversity respectively, both consistently decreased in diversity throughout the experiment and did not produce a stable chemical profile. High diversity within a community is highly correlated with functional redundancy, when multiple community members are capable of the same metabolic process [38]. Diversity is also correlated with functional complementation, when community members differ in metabolic capability [16]. Both functional redundancy and complementation promote stability in a complex community [16,17,39]. Functional complementation is especially important if, as in our case, a complex range of products is desired [40]. Our results indicate that initial inoculum diversity is not sufficient to predict the diversity and production potential of the microbial community that arises from that inoculum within an anaerobic fermentation bioreactor. This contrasts the idea that high inoculum diversity alone is sufficient for ecosystem function [15] as well as the classical idea of given high diversity, “everything is everywhere and the environment selects” [33,34]. While inoculum diversity may outweigh composition for broad ecosystem functions such as respiration and biomass [15] our results suggest that under the conditions tested, inoculum composition plays a significant role in specific ecosystem functions.

**Fig 6:**
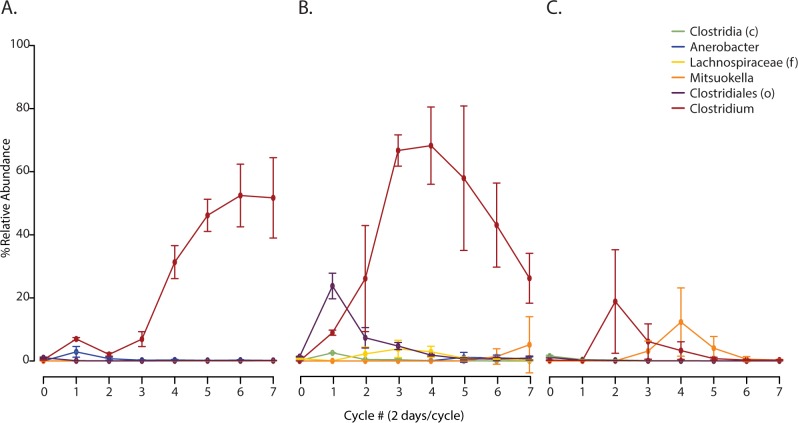
Dynamics of abundant Clostridia species are distinct to starting inoculum. We illustrate changes in average relative abundances of Clostridia OTUs over time in Camel **(A)**, Mangrove **(B)**, and Sludge **(C)** bioreactors. Abundance is represented as average percent relative abundance. OTUs were filtered for equal or greater than 0.1% relative abundance across all time points, and are labeled with the lowest taxonomic classification identified, genus level unless otherwise noted (f, family; o, order; c, class).

The decreased production observed in the Camel and Sludge reactors indicate that key species required for multispecies metabolic pathways may be missing. Bacterial species belonging to the class Clostridia have been shown to be important for the production of VFAs via carboxylate [1] and CoA pathways [31]. Compared to the other inoculum types, Mangrove reactors had higher overall abundances and diversity (Fig 6 and S1 Table) of OTUs classified as Clostridia. It is important to note that the initial Mangrove inoculum, observed as the community profile at cycle zero, did not contain the highest abundance of OTUs classified as Clostridia (S1 Table). This indicates that the Mangrove inoculum may contain the highest number of Clostridia that are resistant to the experimental selection pressures and hints at the large impact strain level differences could have in production capability.

Our results indicate that inoculum source deterministically contributes in shaping community structure and specific ecosystem function. Inoculum-specific outcomes suggest the influence of additional factors such as species-species interactions or variable metabolic gene content [41,42]. While the role of strain level differences cannot be tested with 16S rRNA gene data alone, previous studies have observed differences associated with metabolism and complex ecological interactions when comparing bacterial strains from distinct environments [41,42]. It is also important to note that it is conceivable the Camel and Sludge bioreactor communities are experiencing a slower adaptive period compared to the Mangrove community, or that they are experiencing senescence. Further studies will need be conducted for extended periods of time and incorporate whole-shotgun metagenomics or transcriptomics analysis in order to confirm the growth stage of these communities and the role individual bacterial groups play in the observed differences in reactor function.

## Supporting information

S1 Text Inocula and fermentation methods utilized. (DOC)

S2 Text Sequence processing methods utilized. (DOC)

S3 Text OTU table analysis methods utilized. (DOC)

S4 Text Description of alpha and beta diversity calculations. (DOC)

S5 Text Description of tools and parameters utilized to generate phylogenetic trees. (DOC)

S6 Text Description of statistical analyses utilized. (DOC)

S1 Fig Rarefication plots of species richness. We illustrate the rarefied observed species calculated for each cycle in the Camel **(A)**, Mangrove **(B)**, and Sludge **(C)** bioreactors.
(PDF)

S2 Fig Microbial communities are reproducible and distinct to initial inoculum. Abundances of OTUs are represented as percent relative abundance in Camel (**A**), Mangrove (**B**), and Sludge (**C**) reactors. Taxonomic identification represents the lowest classification identified using the GreenGenes database (version gg_13_5) (DeSantis *et al*., 2006); class (c), family (f), genera (g), species (s). Plot was generated using Qiime (Caporaso *et al*., 2010).
(PDF)

S3 Fig Dynamics of individual OTUs are reproducible across replicates. Abundances are represented as average percent relative abundance of specific OTUs in replicate bioreactors across varying inoculum (replicate 1 = blue, replicate 2 = green, replicate 3 = red). These specific OTUs represent the most abundant OTUs present in bioreactor communities after cycle 1: *Escherichia coli* (**A)**, Enterobacteriaceae (**B)**, *Clostridium acetobutylicum* (**C**), Gammaproteobacteria (**D**), *Clostridium pasteurianum* (**E**), *Bacteroides* (**F**), Prevotella (**G**), Clostridium (**H**), *Vibrio fortis* (**I**), Viellonellaceae (**J**), *Bifidobacterium animalis* (**K**). Points for samples representing Mangrove bioreactor replicate two, cycle one and Sludge bioreactor replicate one, cycle six have been excluded.
(PDF)

S1 Table Results of permutational multivariant analysis performed on microbial community data. We summarize the results obtained for multivariate analysis of within group dispersion (group dispersion) and between group differences (PERMANOVA) as the p-values obtained from these analyses of the microbial community data for each cycle.
(PDF)

S2 Table Results of permutational multivariant analysis performed on chemical profile data. We summarize the results obtained for multivariate analysis of within group dispersion (group dispersion) and between group differences (PERMANOVA) as the p-values obtained from these analyses of the chemical profile data for each cycle. P-values for cycles zero are not included, as chemical data was not collected at the baseline time point.
(PDF)

S3 Table Abundance of Clostridia OTUs in initial inoculum is not predictive of Clostridia abundance in reactor community. Averages represent relative abundance of OTUs belonging to class Clostridia. St. Dev. column represents the standard deviation of the relative abundances across replicate reactors, n = 3.
(PDF)
