# Supplementary material for: Inoculum composition determines microbial community and function in an anaerobic sequential batch reactor
Source: PLoS One. 2017 Feb 14;12(2):e0171369. doi: 10.1371/journal.pone.0171369 (PMC5308813; doi:10.1371/journal.pone.0171369)
Supplement: S1 Text — (DOC) [file pone.0171369.s001.doc]

## S1 Text. Inocula and fermentation methods utilized.

Mangrove, Camel, and Sludge inocula were stored at 4 °C for 43 hours, 24 hours, and 4 days respectively prior to the start of experiments.
