# Supplementary material for: Inoculum composition determines microbial community and function in an anaerobic sequential batch reactor
Source: PLoS One. 2017 Feb 14;12(2):e0171369. doi: 10.1371/journal.pone.0171369 (PMC5308813; doi:10.1371/journal.pone.0171369)
Supplement: S2 Text — (DOC) [file pone.0171369.s002.doc]

**S2 Text. Sequence processing methods utilized.**

Raw data was quality filtered using QIIME (version 1.3.0) (Caporas*o et a*l., 2010). Fastq files were processed using the split_library_fastq.py program of QIIME, truncating sequences when more than one base in a row dropped below Phred quality score 14, corresponding to a probability of error around 3.98% (--last_bad_character N, --max_bad_run_length 1). Only sequences at least 99 bps long after quality filtering was retained (-min_per_read_length 99). All other parameters for this step were default parameters.

Sequences representing OTUs of interest were later reviewed using a minimum length of 150 (-min_per_read_length 150, in Qiime) (Caporas*o et a*l., 2010). These longer sequences, 130 and 127 base pairs after primer removal from the forward and reverse read fastq file, respectively; were used for performing a NCBI BLAST (nt, megablast) searches to better taxonomically identify these OTUs below the genera level, the classification level that the RDP classifier is capable of.
