# Supplementary material for: Inoculum composition determines microbial community and function in an anaerobic sequential batch reactor
Source: PLoS One. 2017 Feb 14;12(2):e0171369. doi: 10.1371/journal.pone.0171369 (PMC5308813; doi:10.1371/journal.pone.0171369)
Supplement: S3 Text — (DOC) [file pone.0171369.s003.doc]

**S3 Text. OTU table analysis methods utilized.**

The filter_samples_from_otu_table.py command in Qiime was used to filter the OTU table by input inoculate and remove samples with less than a thousand read counts from further processing (-n 1000)(Caporas*o et a*l., 2010).
