# Supplementary material for: Inoculum composition determines microbial community and function in an anaerobic sequential batch reactor
Source: PLoS One. 2017 Feb 14;12(2):e0171369. doi: 10.1371/journal.pone.0171369 (PMC5308813; doi:10.1371/journal.pone.0171369)
Supplement: S4 Text — (DOC) [file pone.0171369.s004.doc]

**S4 Text. Description of alpha and beta diversity calculations**. Alpha (Shannon diversity) and beta (Jensen-Shannon distance) diversities were calculated with sample_diversity indices = ['Shannon']) and dist_mat (metric='JS') commands, respectfully, in pysurvey (<https://bitbucket.org/yonatanf/pysurvey>, https://github.com/swo/pysurvey). The Shannon diversity metric used was shannon entropy and is defined as H =  - Sum_i_ (p_i_ * log p_i_), with p_i_ being the fraction of OTU_i_.
