# Supplementary material for: Inoculum composition determines microbial community and function in an anaerobic sequential batch reactor
Source: PLoS One. 2017 Feb 14;12(2):e0171369. doi: 10.1371/journal.pone.0171369 (PMC5308813; doi:10.1371/journal.pone.0171369)
Supplement: S5 Text — (DOC) [file pone.0171369.s005.doc]

**S5 Text. Description of tools and parameters utilized to generate phylogenetic trees.** Aligned sequences used for tree building were trimmed of gaps using trimAL (version 1.2) with a gap threshold of 0.05 so gaps present in 95% or more of the sequences would be removed (-gt 0.05)(27). FastTree (28) was then used to build a phylogenetic tree from the trimmed sequences using nucleaotide alignment (-nt), a GTR+CAT model (-gtr), and constraint file created from RDP classifications described above (-constraint). The constraint file was generated using custom a Python script (Python 2.7.10).
