# Supplementary material for: Inoculum composition determines microbial community and function in an anaerobic sequential batch reactor
Source: PLoS One. 2017 Feb 14;12(2):e0171369. doi: 10.1371/journal.pone.0171369 (PMC5308813; doi:10.1371/journal.pone.0171369)
Supplement: S6 Text — (DOC) [file pone.0171369.s006.doc]

**S6 Text. Description of statistical analyses utilized.** The adonis function of the vegan package (version 2.4-1) in R (version 3.2.3) was utilized to perform permutational multivariate analysis to assess the significance of differences in chemistry and microbial composition between the inoculum sources. The functions permutest and betadisper in the R package vegan (version 2.4-1) were utilized to perform multivariate homogeneity of group dispersion to assess the significance of variance between replicates of the different inoculum sources. 999 permutations were used for both multivariate analyses.
