# Supplementary figures and images for: Inoculum composition determines microbial community and function in an anaerobic sequential batch reactor
Source: PLoS One. 2017 Feb 14;12(2):e0171369. doi: 10.1371/journal.pone.0171369 (PMC5308813; doi:10.1371/journal.pone.0171369)

A.

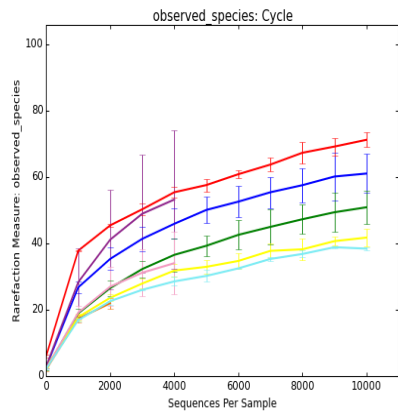

B.

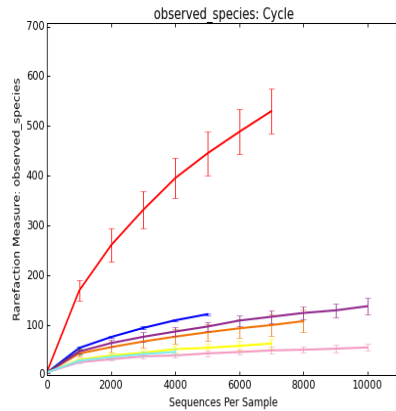

C.

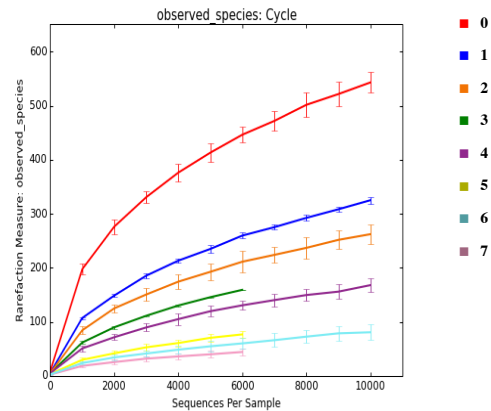

Supplement: S1 Fig — We illustrate the rarefied observed species calculated for each cycle in the Camel (A), Mangrove (B), and Sludge (C) bioreactors. (PDF) [file pone.0171369.s007.pdf]

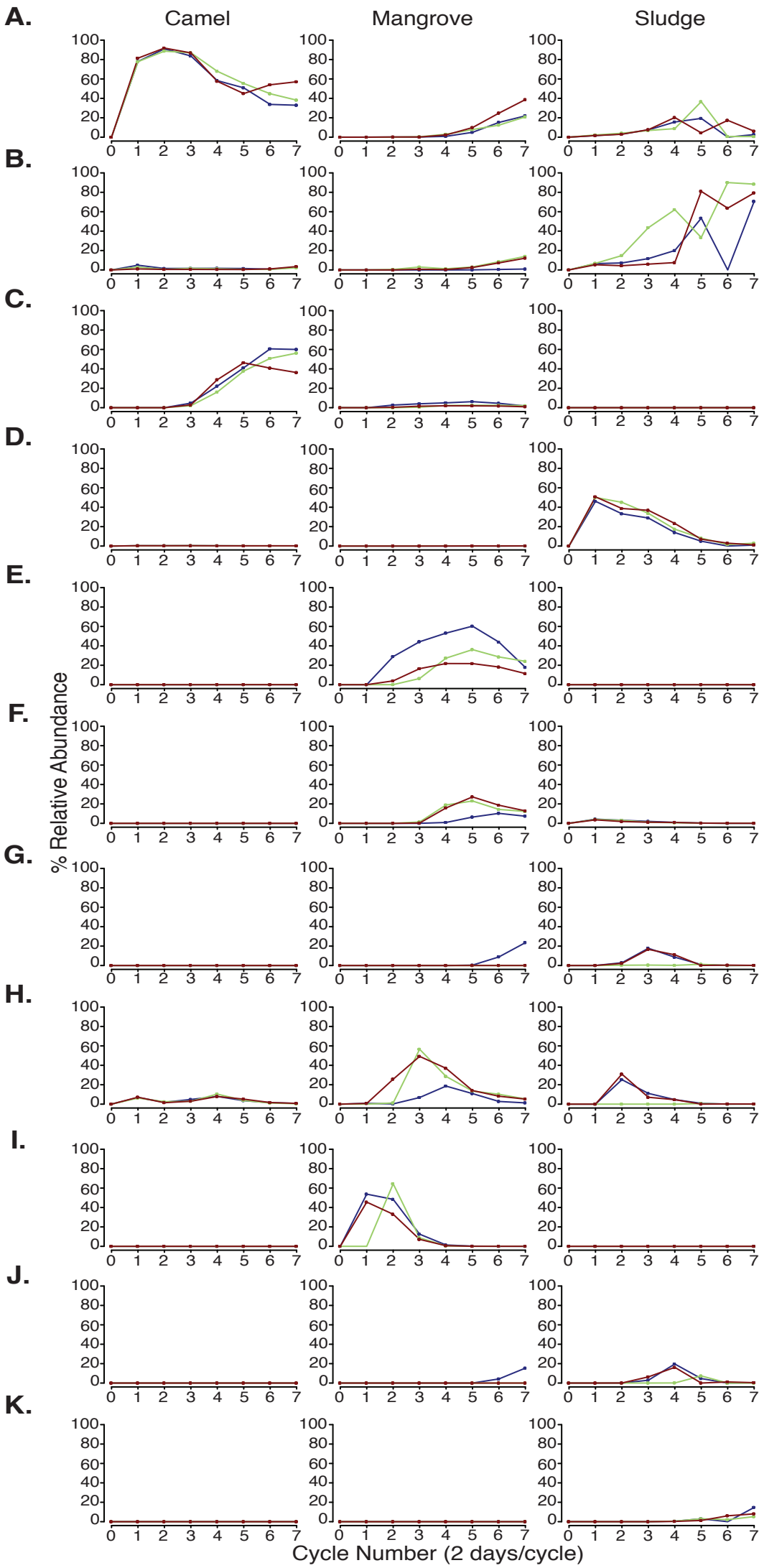

Supplement: S3 Fig — Abundances are represented as average percent relative abundance of specific OTUs in replicate bioreactors across varying inoculum (replicate 1 = blue, replicate 2 = green, replicate 3 = red). These specific OTUs represent the most abundant OTUs present in bioreactor communities after cycle 1: Escherichia coli (A), Enterobacteriaceae (B), Clostridium acetobutylicum (C), Gammaproteobacteria (D), Clostridium pasteurianum (E), Bacteroides (F), Prevotella (G), Clostridium (H), Vibrio fortis (I), Viellonellaceae (J), Bifidobacterium animalis (K). Points for samples representing Mangrove bioreactor replicate two, cycle one and Sludge bioreactor replicate one, cycle six have been excluded. (PDF) [file pone.0171369.s009.pdf]
