# Supplementary material for: Inoculum composition determines microbial community and function in an anaerobic sequential batch reactor
Source: PLoS One. 2017 Feb 14;12(2):e0171369. doi: 10.1371/journal.pone.0171369 (PMC5308813; doi:10.1371/journal.pone.0171369)
Supplement: S2 Fig — Abundances of OTUs are represented as percent relative abundance in Camel (A), Mangrove (B), and Sludge (C) reactors. Taxonomic identification represents the lowest classification identified using the GreenGenes database (version gg_13_5) (DeSantis et al., 2006); class (c), family (f), genera (g), species (s). Plot was generated using Qiime (Caporaso et al., 2010). (PDF) [file pone.0171369.s008.pdf]

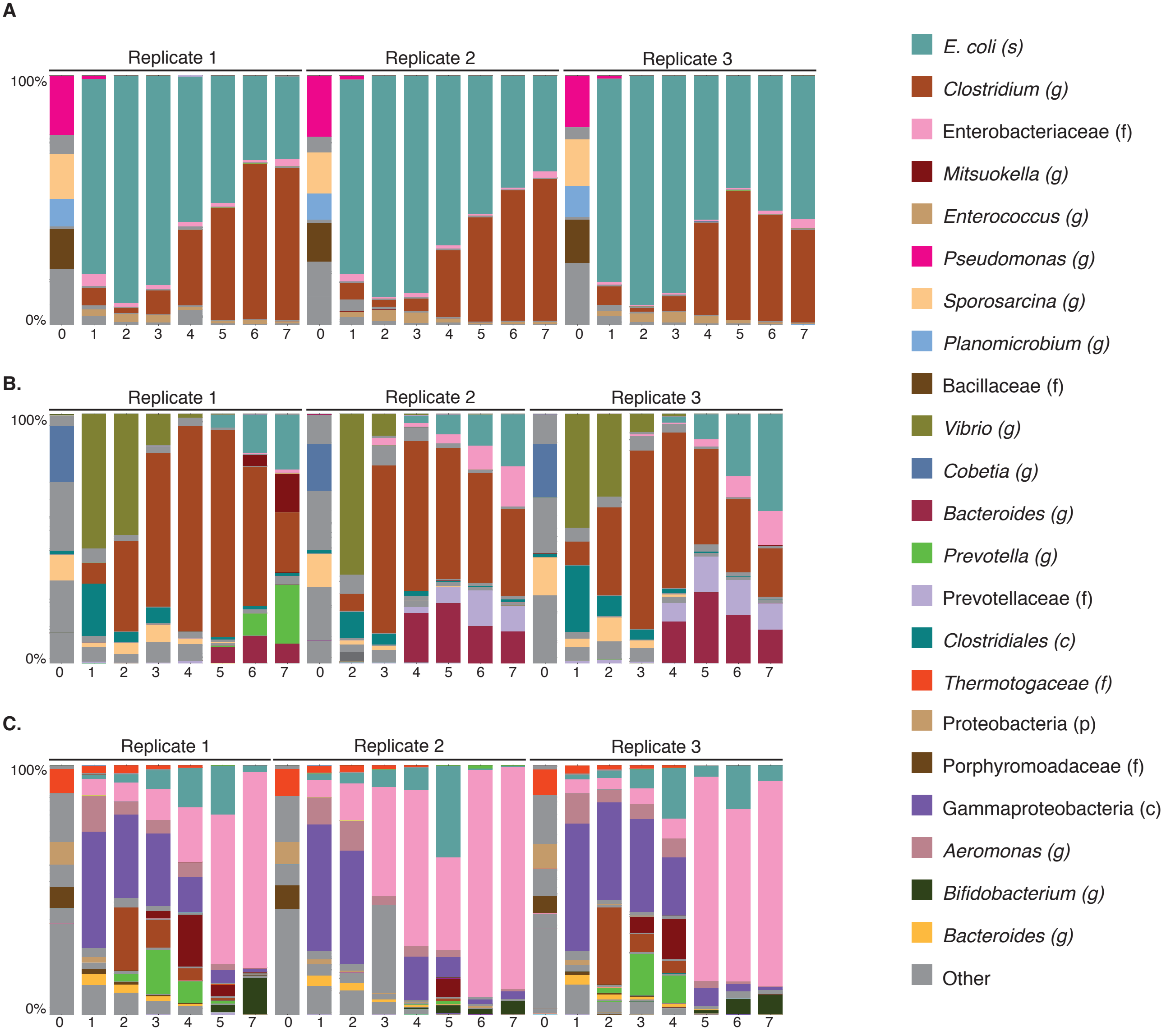

A
Replicate 1
Replicate 2
Replicate 3
100%
0%
0 1 2 3 4 5 6 7 0 1 2 3 4 5 6 7 0 1 2 3 4 5 6 7
B.
Replicate 1
Replicate 2
Replicate 3
100%
0%
0 1 2 3 4 5 6 7 0 2 3 4 5 6 7 0 1 2 3 4 5 6 7
C.
Replicate 1
Replicate 2
Replicate 3
100%
0%
0 1 2 3 4 5 7 0 1 2 3 4 5 6 7 0 1 2 3 4 5 6 7
E. coli (s)
Clostridium (g)
Enterobacteriaceae (f)
Mitsuokella (g)
Enterococcus (g)
Pseudomonas (g)
Sporosarcina (g)
Planomicrobium (g)
Bacillaceae (f)
Vibrio (g)
Cobetia (g)
Bacteroides (g)
Prevotella (g)
Prevotellaceae (f)
Clostridiales (c)
Thermotogaceae (f)
Proteobacteria (p)
Porphyromoadaceae (f)
Gammaproteobacteria (c)
Aeromonas (g)
Bifidobacterium (g)
Bacteroides (g)
Other
