# Supplementary material for: Inoculum composition determines microbial community and function in an anaerobic sequential batch reactor
Source: PLoS One. 2017 Feb 14;12(2):e0171369. doi: 10.1371/journal.pone.0171369 (PMC5308813; doi:10.1371/journal.pone.0171369)
Supplement: S1 Table — We summarize the results obtained for multivariate analysis of within group dispersion (group dispersion) and between group differences (PERMANOVA) as the p-values obtained from these analyses of the microbial community data for each cycle. (PDF) [file pone.0171369.s010.pdf]

Cycle	Group Dispersion p-value	Permanova p-value
0	0.35	0.005
1	0.09	0.005
2	0.14	0.001
3	0.1	0.004
4	0.24	0.005
5	0.09	0.004
6	0.01	0.003
7	0.35	0.005
