# Supplementary material for: Inoculum composition determines microbial community and function in an anaerobic sequential batch reactor
Source: PLoS One. 2017 Feb 14;12(2):e0171369. doi: 10.1371/journal.pone.0171369 (PMC5308813; doi:10.1371/journal.pone.0171369)
Supplement: S2 Table — We summarize the results obtained for multivariate analysis of within group dispersion (group dispersion) and between group differences (PERMANOVA) as the p-values obtained from these analyses of the chemical profile data for each cycle. P-values for cycles zero are not included, as chemical data was not collected at the baseline time point. (PDF) [file pone.0171369.s011.pdf]

Cycle	Group Dispersion p-value	Permanova p-value
0	-	-
1	0.62	0.005
2	0.62	0.013
3	0.42	0.03
4	0.54	0.003
5	0.47	0.003
6	0.15	0.001
7	0.1	0.005
