# Supplementary material for: Inoculum composition determines microbial community and function in an anaerobic sequential batch reactor
Source: PLoS One. 2017 Feb 14;12(2):e0171369. doi: 10.1371/journal.pone.0171369 (PMC5308813; doi:10.1371/journal.pone.0171369)
Supplement: S3 Table — Averages represent relative abundance of OTUs belonging to class Clostridia. St. Dev. column represents the standard deviation of the relative abundances across replicate reactors, n = 3. (PDF) [file pone.0171369.s012.pdf]

	Camel		Mangrove		Sludge	
Cycle	Average	St. Dev.	Average	St. Dev.	Average	St. Dev.
0	0.02	0	0.02	0	0.06	0.01
1	0.1	0.01	0.36	0.02	0.02	0
2	0.03	0.01	0.37	0.1	0.2	0.16
3	0.07	0.02	0.76	0.04	0.1	0.08
4	0.32	0.04	0.73	0.09	0.16	0.14
5	0.47	0.04	0.61	0.18	0.06	0.04
6	0.53	0.08	0.47	0.12	0.01	0.01
7	0.52	0.1	0.34	0.09	0.01	0
